# University marathons as structured health interventions: deconstructing the role of event culture, organization, activities and services in student engagement

**DOI:** 10.3389/fspor.2026.1763710

**Published:** 2026-05-14

**Authors:** Yongsheng Lin, Jinwen Xie, Fugao Jiang, Liangxingyun He

**Affiliations:** 1 School Sports Science, Qufu Normal University, Jining, China; 2 Department of Physical Education, Xiamen University of Technology, Ximen, China; 3 Stockholm School of Economics, Stockholm, Sweden

**Keywords:** campus sports culture, health promotion, sport for education, structural equation modeling, university marathon, value co-creation

## Abstract

**Background:**

As an integral component of the higher education ecosystem, sport serves as a critical vehicle for fulfilling a university's educational mission and cultivating its institutional culture. With the rise of marathons on university campuses, the core challenge of event branding shifts from the economic pursuits of city marathons to the pedagogical imperatives of the university's “Sport for Education mission”. However, the formative mechanism of this unique brand value remains empirically under explored. This study addresses this gap by examining a professional-grade university marathon in Southern China.

**Methods:**

Utilizing Structural Equation Modeling (SEM), we analyzed data from 764 participants in a World Athletics-certified university marathon in Southern China. A four-dimensional framework—Event Activities, Organization, Culture, and Services—was constructed to evaluate causal impacts on engagement.

**Results:**

The analysis validated a hierarchical pathway (x^2^/df < 5, RMSEA < 0.08). Event Activities (0.55) drive engagement, stabilized by Organization (0.45) and elevated by Culture (0.42). Crucially, Event Services (0.35) facilitate value co-creation, securing participant loyalty.

**Conclusion:**

University marathons function as dynamic psychosocial ecosystems. The validated Activity-Organization-Culture-Service chain provides a robust framework for translating educational missions into tangible physical activity adherence and campus social cohesion.

## 1 Introduction

As an integral component of the higher education ecosystem, sport serves as a critical vehicle for fulfilling a university's educational mission and cultivating its institutional culture. Beyond the simple pursuit of physical fitness, university-based sporting events function as structured temporal and spatial opportunities for fostering holistic student development, encompassing character building, resilience, and social cohesion. Beyond the simple pursuit of physical fitness, university-based sporting events function as structured temporal and spatial opportunities for fostering holistic student development, with their educational connotations encompassing character building, resilience cultivation, and social cohesion development. Recent studies have demonstrated that outdoor campus physical activities can attenuate academic burnout among Chinese university students through dual pathways: the direct physiological pathway and the cultivation of nature connectedness, and that the formation of sustained sports participation habits can significantly strengthen individuals' social identity and emotional belonging (1, 2).Within this educational framework, the university marathon has emerged as a unique sub-domain, distinct from the commercially driven city marathon (3, 4). Unlike urban events that prioritize economic impact and destination branding, the university marathon is deeply rooted in the pedagogical imperative of “Sport for Education”. From a brand architecture perspective, such events are not standalone commercial entities but strategic sub-brand extensions of the university itself. Their primary function is to activate and disseminate the institution's core values—transforming abstract educational philosophies into tangible, embodied experiences for the student body.

However, the rapid proliferation of these events has outpaced our theoretical understanding of their internal value-generation mechanisms. While existing literature has extensively covered operational aspects such as athletic training feasibility, cultural construction, and risk management, a critical theoretical gap persists (5–11). Specifically, the mechanism through which a university's abstract educational mission is translated into concrete event brand value remains empirically underexplored. We lack a clear understanding of how event activities, organization, and culture interact to transform a physical race into a “lived” educational brand that fosters loyalty and value co-creation. The dynamics of how students perceive and internalize the “Sport for Education” promise through these specific event dimensions have not been fully deconstructed.

To address this void, this study employs Structural Equation Modeling (SEM) to deconstruct the brand value generation mechanism of a professional-grade university marathon in Southern China. Drawing on survey data from 764 student participants, this research validates a four-stage, logically progressive path: beginning with experiential Event Activities, solidified by professional Event Organization, elevated by distinctive Event Culture, and culminating in Event Services that foster value co-creation. By clarifying these causal relationships, this study provides empirical evidence for the application of brand theory in mission-driven organizations and offers a robust analytical framework for university administrators to strategically leverage sporting events as instruments of holistic student development.

## 2 Literature review and hypotheses development

### 2.1 Literature review

The brand construction of a university marathon represents the systematic development of a cultural product that carries the core mission of “Sport for Education” within the unique ecosystem of higher education. This process is not a simple transplantation of commercial marketing theories but demands an integrated theoretical framework that connects strategic intent to experiential reality. This study's framework is grounded in classic brand theory, progressively analyzing the logic of university marathon brand building through the four dimensions of brand positioning, brand ontology and identity, and brand promise.

#### 2.1.1 Brand positioning theory

Brand positioning begins with a product but aims to secure a unique and valuable place in the mind of the prospective consumer (12, 13). This mental real estate can be claimed at three levels, attribute, benefit, and value (14). For a university marathon, positioning must transcend the basic attribute level (e.g., “the only B-label certified campus race in Asia”) or the benefit level (e.g., “a platform for physical fitness and social networking”). Its soul lies at the value level, where the event is inextricably linked to participants' deep-seated values and the university's core mission. The brand's positioning is not about differentiating itself in a commercial race market but about establishing its irreplaceable “Sport for Education” value proposition amidst a crowded field of campus activities. However, an abstract positioning is merely a castle in the air without a tangible experiential carrier. In this context, Event Activities serve as the most direct and essential vehicle for delivering and validating this educational value proposition. The race itself, pre-race training camps, and sports health seminars collectively constitute the brand's “core product”, serving as the critical touch-points where the brand's positioning is operational and its promise is initially fulfilled.

#### 2.1.2 Brand ontology and identity theory

Brand ontology theory moves beyond an instrumental view to explore the brand's cultural soul and intrinsic substance. As Wang posits, a brand is more than a product identifier; it possesses its own content, with the physical product serving merely as a vessel for specific cultural meanings (15). The “ontology” of a university marathon is not a mere athletic competition but a modern cultural ritual designed to shape character and build community. It embodies the spirit of self-challenge, the pursuit of excellence, and perseverance (16). A semiotic framework helps to clarify this, suggesting that a brand's coherence is achieved by calibrating the gaps between its brand ontology (the core, abstract essence—the “Sport for Education” mission), its brand types (recurring symbols and signifiers like logos and slogans), and its brand tokens (the physical instantiations participants interact with, such as a finisher's medal). This intangible essence requires a tangible symbolic system for its materialization and dissemination. Brand identity theory provides this lens, viewing a brand as a complex symbol system that uses logos, colors, and slogans to establish emotional connections with consumers (17). For the university marathon, the dimension of Event Culture represents the organic synthesis of brand ontology and identity. It encompasses both the intrinsic sporting spirit and the materialized cultural symbols (e.g., medals incorporating the university crest, slogans reflecting the school motto) and ritualized atmosphere. Through these cultural symbols and rituals, the abstract concept of education is transformed into profound personal memories and a sense of collective identity for participants.

#### 2.1.3 Brand promise theory

After defining the brand's strategic proposition and cultural embodiment, it is necessary to introduce a core theory to explain how brand value is reliably delivered to establish a lasting relationship of trust. This is the Theory of Brand Promise. In their seminal study, Brand as Promise, scholars Bhargava and Bedi conducted a profound ethical reconstruction of this theory. They point out that the traditional view of a brand as a collection of shared associations cannot explain why a brand's breach of trust triggers intense moral indignation from consumers. To address this, they propose that a brand should be viewed as a series of normatively binding expectations that are ethically equivalent to a promise, serving as the bridge connecting brand intent with consumer experience (18). This implies that when a brand fails to deliver on its promise, it is perceived by consumers not merely as a service failure but as a breach of contract, which can precipitate a crisis of trust in the brand.

This theory reveals the mechanism by which brand trust is generated, the core of which stems from the individual's belief that the brand can fulfill its value commitment. The consistent and reliable fulfillment of promises is the sole path to building brand trust, and trust, in turn, is the critical prerequisite for brand loyalty. In the context of a campus marathon, holistic development through athletics is its most central brand promise. Existing research has confirmed that event organization can enhance the quality of a marathon and its level of public recognition (19), and that event services are a decisive factor influencing participants' perception of the event brand (6). Therefore, this study integrates the two dimensions of event organization and event services into the brand-building pathway for a campus marathon. It treats event services and organization as the “delivery system” for the core brand promise, rather than as mere logistical support functions. These two elements transform the abstract concept of holistic development into a safe, reliable, and caring tangible experience. They are fundamental to establishing the brand's credibility and fostering participant loyalty, constituting the most solid cornerstone of brand equity.

In summary, the theoretical framework of this study is not a patchwork of existing brand theories but rather an integrated and reconstructed brand-building framework with an inherent logical progression, specifically adapted for the campus marathon context (Table 1). This model posits that the value generation of a successful campus marathon brand follows a clear path: it begins with a distinct strategic proposition, holistic development through athletics, established by the theory of brand positioning. Subsequently, this proposition is endowed with profound cultural embodiment, internalized as participants' emotional identification through symbols and rituals guided by brand ontology and brand identity theories. Ultimately, all of this must be guaranteed by solid operational credibility. According to the Theory of Brand Promise, this is achieved by reliably delivering on the promise and building trust through professional event organization and human-centered event services. Consequently, the four dimensions proposed by this study—event activities, event culture, event organization, and event services—constitute a complete value creation chain, from brand positioning and cultural empowerment to promise fulfillment.

**Table 1:** Brand theory and empirical connection.

Theoretical dimension	Core concept	Connection to university marathon brand building	Empirical dimension
Brand positioning theory	Value proposition	The brand's value proposition is delivered and validated through the core experiential carrier of event activities.	Event activities
Brand ontology & identity theory	Cultural embodiment	The brand's spiritual and cultural essence is translated into participant's emotional identification and collective memory through symbols (medals, logos) and rituals (atmosphere, the challenge itself).	Event culture
Brand promise theory	Promise fulfillment & brand loyalty	The brand promise is fulfilled through a delivery system of professional organization and humanistic services, thereby establishing brand credibility and participant loyalty.	Event organization, event services

### 2.2 Hypotheses development

(1) Event Culture. Brand ontology and identity theories emphasize that a brand's intrinsic essence and cultural soul are central to establishing a deep emotional connection with its audience, forming a stable and unified perception. Event culture, comprising material, institutional, behavioral, and spiritual dimensions (20), collectively shapes the event's overall image and brand value. The value of this culture lies not in its static symbols (e.g., logos, slogans) but in its dynamic function as a social process that catalyzes and solidifies participants' community identity. The university marathon provides an ideal setting for this, as participants face challenges, encourage one another, and share success, reinforcing their common identity as members of the university community. Cultural symbols, such as medals featuring the university crest or slogans embodying the school motto, become markers of this collective identity. When a participant earns a medal through personal effort, they receive not just a souvenir but a symbol of an “earned identity”. This process elevates the event from a mere athletic activity to a communal practice rooted in the university's cultural fabric, forging the deepest emotional bonds between the brand and its participants. Therefore, the following hypothesis is proposed:
  H1: Event Culture is a significant positive predictor of university marathon brand building.
(2) Event Organization. Event branding theory regards the “event product” and its delivery process as the ultimate carriers of brand value. Bhargava et al. reframe the brand itself as a set of normatively binding expectations made to the consumer, ethically akin to a promise. Consequently, meticulous risk management, clear communication, reliable on-site execution, and professional volunteer services collectively form the “delivery system” for this brand promise (18). This system directly influences participants' overall perception of the event's quality and ultimately their propensity for brand loyalty (21). In the unique context of a university, when students perceive the fulfillment of the “Sport for Education” promise through well-executed event-related activities, their trust in the brand significantly increases. The various volunteers teams, media, logistics serve as the personification of this commitment, and their service quality directly impacts the participant experience. Thus, event organization is not only a safeguard for the brand promise but also the cornerstone for building enduring brand trust. Therefore, the following hypothesis is proposed:
  H2: Event Organization is a significant positive predictor of university marathon brand building.
(3) Event Activities. According to brand positioning theory, the primary task of brand building is to occupy a unique and appealing position in the minds of the target audience. For a university marathon, the most central and direct “product” is the event's activities. These activities are a fundamental and widespread manifestation of campus sports culture (22) and a primary extension of classroom-based physical education. A rich and creative system of activities can transform the abstract concept of “Sport for Education” into a tangible, embodied experience for participants that the first and most critical step in brand engagement. This experience must align with the intrinsic motivations of the target audience. Research indicates that participants in endurance sports are often motivated by the pursuit of personal achievement, health and wellness concerns, and the release of psychological stress (23, 24). Therefore, the design of the university marathon experience should not be limited to the race itself but should encompass a matrix of activities sensory, behavioral, emotional, and cognitive built around these core motivations. An attractive event that meets the intrinsic needs of participants is the absolute prerequisite for stimulating initial engagement and forming a positive first impression of the brand. Therefore, the following hypothesis is proposed:
  H3: Event Activities is a significant positive predictor of university marathon brand building.
(4) Event Services. In brand theory, a positive customer experience is a core component in building brand satisfaction and enhancing brand equity (25). High-quality event services, from pre-race registration inquiries to in-race aid and medical support to post-race results access, directly shape the participant's overall event experience (26, 27). In the university marathon context, the organizer's role evolves from a mere “service provider” to an “enabler of a value co-creation platform”. By providing various service touchpoints (e.g., user-friendly online registration, real-time information apps, aid stations, medical tents), they create the conditions for interaction among participants and between participants and the event itself. Participants, in turn, actively engage in shaping the event experience and brand narrative through their participation, social media sharing, and mutual encouragement on the course, becoming co-creators of value. A well-designed service system should maximally empower participants, transforming them from passive recipients of service into active co-builders of the brand. This process converts fleeting event satisfaction into lasting brand loyalty and advocacy. Therefore, the following hypothesis is proposed:
  H4: Event Services is a significant positive predictor of university marathon brand building.

## 3 Methodology

### 3.1 Research context and data collection

This study selected the university marathon hosted by Xiamen Institute of Technology, located in Southern China, as its empirical case. This event is currently the only university marathon in Asia to have received a World Athletics B-label road race certification, making its high degree of professionalism and mature brand foundation an ideal sample for testing the proposed theoretical model.

Data were collected on-site during the autumn race on November 18, 2023. The research team employed a random distribution strategy, administering paper-based questionnaires at multiple locations, including post-race rest areas, sponsor activity zones, dining hall entrances, student dormitories, and athlete service areas. To mitigate potential sampling bias for instance, over-representing highly engaged runners in the post-race rest area the team implemented two key strategies. First, the demographic section of the questionnaire was designed to distinguish participant roles and activity types, allowing for the isolation of data from student runners. Second, a pre-test was conducted to assess and adjust the questionnaire distribution ratios across different locations, ensuring broad coverage of participants with varying levels of engagement.

A total of 900 questionnaires were distributed and fully recovered. After excluding responses from non-student participants, external personnel, volunteers, support staff, media teams, and community runners, 764 valid samples were obtained, resulting in an effective response rate of 84.9%. All data were collected anonymously, and the entire research process received approval from the event organizing committee and the ethical review board of the research team's institution.

### 3.2 Measurement instrument

The survey instrument consisted of four main sections. The measurement items were primarily adapted from validated scales in existing literature and modified to fit the specific context of a university marathon. Items for Event Culture were adapted from Dang and Wang, originally comprising 9 indicators. Items for Event Organization were based on Ke, with 5 indicators. Event Activities were measured using 4 indicators adapted from Markovits, and Event Services were assessed with 9 indicators from Huang. The wording of the items was further refined based on the results of a pre-survey to create the final questionnaire (28–32).

## 4 Results

This study employed a quantitative approach using survey data, analyzed with SPSS 29.0 and Amos 26.0. The analysis proceeded in three stages. First, the reliability and validity of the measurement scales were assessed. Second, Exploratory Factor Analysis (EFA) with varimax rotation was used to extract and name common factors, establishing a university marathon brand-building evaluation scale. Finally, the structural model was constructed and tested using Confirmatory Factor Analysis (CFA) to evaluate its fit with the data and to test the research hypotheses.

### 4.1 Measurement model assessment: reliability and validity

#### 4.1.1 Exploratory factor analysis

To ensure the data's suitability for Structural Equation Modeling (SEM), descriptive statistics for each variable, including standard deviation, skewness, and kurtosis, were first examined using SPSS 29.0. The standard deviations ranged from 0.472 to 0.861, indicating a moderate level of data dispersion. Skewness values were generally within the acceptable range of ±2 (with the lowest being −1.918 for item X1), suggesting no extreme skewness. Kurtosis values were mostly within the acceptable range of ±7, with only item X8 showing a value of 8.287, indicating a somewhat peaked or heavy-tailed distribution. However, this value was still considered statistically acceptable for further analysis (33).

The Kaiser-Meyer-Olkin (KMO) measure of sampling adequacy was 0.864, well above the recommended threshold of 0.8, indicating that the data were suitable for EFA. Bartlett's test of sphericity was significant (approximate Chi-square = 15,143.992, df = 300, *p* < 0.001), confirming that the correlation matrix was not an identity matrix and that factor analysis was appropriate (Table 2).

**Table 2:** KMO and Bartlett's test results.

Kaiser-Meyer-Olkin measure of sampling adequacy		0.864
Bartlett's test of sphericity	Approx. Chi-Square	15,143.992
	df	300
	Sig.	0.000

Using principal component analysis with varimax rotation, factors were extracted, retaining indicators with absolute values greater than 0.5. Two items, “Alignment of event spirit with school motto” (0.440) and “Attention to various rituals in event activities” (0.410), were removed due to low loadings. The final analysis yielded four factors with a total of 25 indicators (Table 3). These factors were named: Event Culture (X1–X7), Event Organization (X8–X12), Event Activities (X13–X17), and Event Services (X18–X25). The Cronbach's alpha coefficients for all factors exceeded the standard threshold of 0.7, indicating good internal consistency and reliability. The cumulative variance explained by the four factors was 69.8%, suggesting that the constructed university marathon brand-building scale is acceptable.

**Table 3:** Reliability and validity of the measurement model.

Latent variable	Item	Stan-dared load	Cronba-ch's alpha	CR	AVE	Average Value	
Event culture	Event Logo	0.649	0.910	0.897	0.565	4.21	4.02
	Event Medals and Trophies	0.668				4.16	
	Event Mascots and Derivative Products	0.568				3.70	
	Event Themes and Slogans	0.538				3.90	
	Facing difficulties with perseverance and challenge	0.948				4.05	
	Cultivating adaptability to society	0.892				4.10	
	Enhancing personal confidence	0.884				4.02	
Event organizational	Competition Management Department	0.572	0.882	0.876	0.597	3.47	3.68
	Sports Club Department	0.857				3.67	
	Logistics Support Department	0.929				3.70	
	Volunteer Service Department	0.872				4.02	
	Media and Publicity Department	0.549				3.55	
Event activities	Organizing sports knowledge and experience sharing	0.955	0.886	0.889	0.670	3.30	3.65
	Conducting quality physical education classes	0.810				3.76	
	Sports and cultural activities	0.782				4.12	
	Regular organization of relevant sports training sessions	0.706				3.45	
Event services	Participating equipment	0.895	0.940	0.935	0.622	3.96	3.93
	Race track replenishment	0.910				4.01	
	Medical emergency services	0.875				3.87	
	Racecourse services	0.798				3.93	
	Official pacers	0.767				3.60	
	Event information dissemination	0.819				3.99	
	Sports data processing	0.677				3.87	
	Online registration	0.536				4.27	
	Sports app	0.748				3.89	

#### 4.1.2 Confirmatory factor analysis

The validity of the measurement model was assessed using Amos 26.0. The criteria for convergent validity were: standardized factor loadings >0.5, composite reliability (CR) >0.6, and average variance extracted (AVE) >0.5. As shown in Table 3, all factor loadings ranged from 0.538 to 0.955, CR values ranged from 0.876 to 0.935, and AVE values ranged from 0.565 to 0.670. These results meet the established standards for validity in SEM (33).

To assess discriminant validity, the Fornell-Larcker criterion was applied. This criterion is met if the square root of a construct's AVE is greater than its correlation coefficients with all other constructs. As shown in Table 4, the square root of the AVE for each latent variable (on the diagonal) is greater than the inter-construct correlations, indicating good discriminant validity among the four variables.

**Table 4:** Discriminant validity of the measurement model.

Latent variable	Event culture	Event organization	Event activities	Event services
Event culture	0.752			
Event organization	0.069***	0.773		
Event activities	0.124***	0.160***	0.819	
Event services	0.079***	0.080***	0.162***	0.788

Diagonal elements (in bold) are the square roots of the Average Variance Extracted (AVE). Off-diagonal elements are the correlations between constructs.

### 4.2 Structural model assessment and hypothesis testing

The sample data were fitted to the structural model, which was successfully identified. A goodness-of-fit test was then conducted. As shown in Table 5, all fit indices met the recommended standards for social science research, indicating that the proposed structural model provides a good fit for the data.

**Table 5:** Structural model Fit indices and path coefficients.

Fit indices	X^2^/DF	RMSEA	RFI	NFI	IFI	TLI	CFI	PNFI	PCFI
Standard	<5	<0.08	>0.9	>0.9	>0.9	>0.9	>0.9	>0.5	>0.5
Fit indices	4.187	0.065	0.918	0.928	0.945	0.936	0.944	0.811	0.825

The standardized regression weights (path coefficients) for the four dimensions, ranked from highest to lowest, were: Event Activities (0.55), Event Organization (0.45), Event Culture (0.42), and Event Services (0.35). All regression weights exceeded 0.30, a common threshold indicating practical significance. Path coefficients above 0.50 are considered to represent a strong contribution (34). Therefore, all four research hypotheses were supported, demonstrating that these dimensions are core drivers of the university marathon's brand equity. The subsequent discussion will analyze these dimensions in descending order of their path coefficients to explore their respective contributions and identify areas for improvement.

## 5 Discussion

### 5.1 Event activities: the core matrix of brand experience

The dimension of Event Activities exhibited the strongest path coefficient (0.55), confirming its critical role as the foundational prerequisite for brand building. From a theoretical standpoint, event activities should not be viewed as a collection of isolated items but as an integrated matrix of brand touchpoints. For a participatory event, brand value cannot exist independently of the participant's direct, embodied experience within the core activities. Every touchpoint, from the race itself to ancillary experiential activities and knowledge-sharing sessions, is a crucial moment where participants interact with the brand and form perceptions. The sum of these interactions constitutes the overall brand experience, which is the central mechanism through which brand value is constructed in the consumer's mind.

The empirical data show high participant agreement with “campus sports culture activities” (M = 4.12) and “offering sufficient and high-quality PE classes” (M = 3.76). This indicates that the event successfully creates strong sensory experiences (e.g., scenic campus course, enthusiastic atmosphere) and behavioral experiences (e.g., the sense of accomplishment from running). These embodied experiences form the foundation of brand appeal, are the most intuitive and easily perceived part of the brand promise, and are also one of the influencing factors for participants to choose to participate again (35).

However, the data also reveals a notable shortcoming in the implementation of supporting activities for campus events: participants have the lowest perceived awareness of “sports knowledge and experience sharing sessions” (M = 3.30). From a longitudinal historical perspective, since China's Spring and Autumn and Warring States periods (770–221 BCE), Confucianism has prevailed, and treating others with ritual propriety has become the behavioral standard for a “Junzi (noble person)” a tradition that has endured for millennia (36). During this period, sports activities were not valued in society, and the notion of “prioritizing literary and academic pursuits over martial and physical endeavors” permeated ancient Chinese society and continues to exert influence today (37).

From the perspective of brand theory, however, this study argues that this phenomenon is not a legacy of the aforementioned “literary-over-physical” ideology (a cultural tradition that prioritizes literary/academic pursuits over martial/physical ones) as traditional views suggest. Instead, it should be interpreted as an insufficiency in the brand's design of touchpoints within the experiential dimension. In the age of the experience economy, successful brands not only provide opportunities for physical participation but also stimulate intellectual resonance among participants (25). For campus marathons, where college students are the primary participants, these individuals are not merely runners but also seekers of knowledge.

The current event design may fail to effectively integrate sports practice with higher-level cognitive needs such as knowledge exploration and skill enhancement. This not only limits the depth and integrity of the brand experience but also undermines the fulfillment of its mission of using sports for education. This indicates that organizers may have missed a valuable opportunity to elevate the event from a purely sports activity to a comprehensive educational platform. This interpretation finds additional empirical traction from Wang et al. SEM analysis, which reveals that exercise-related self-efficacy partially mediates the relationship between perceived event attractiveness and participation intention—suggesting that knowledge-sharing sessions may function not merely as informational touchpoints but as catalysts for building participants' sport-specific confidence and sustaining long-term brand engagement.

### 5.2 Event organization: the cornerstone of brand trust and promise delivery

Once this experiential foundation is established through compelling activities, the reliability of the Event Organization becomes the critical factor in converting initial positive sentiment into enduring brand trust. With a path coefficient of 0.45, Event Organization ranks second in importance, highlighting its crucial role in converting initial positive experiences into enduring brand trust. This finding aligns perfectly with brand promise theory, which posits that the university marathon makes an implicit promise of a safe, professional, and excellent experience to its participants. Efficient and reliable organization is the key mechanism for delivering on this promise.

The empirical data provide concrete support for this theoretical link. The event committee at Xiamen Institute of Technology, with its specialized departments for competition management, logistics, and volunteer services, is not a hollow administrative structure but a highly professionalized and refined organizational matrix. Each department has clear responsibilities, collectively forming the delivery system for the brand promise. The Competition Management Department ensures professionalism and compliance by liaising with athletic associations, while the Logistics Support Department translates the promise of safety into tangible reality through meticulous course risk assessments and medical contingency plans. The Volunteer Services Department, through systematic professional training, ensures the quality and warmth of every service touchpoint.

The effectiveness of this systematic organization is reflected not only in the strong path coefficient but also in the micro-level data. The “Volunteer Services Department” received the highest mean score within this dimension (4.02). Volunteers are the human embodiment of the brand promise. Their professionalism and enthusiasm are perceived not merely as “good service” but as tangible proof of the organizer's concern for participant well-being. This perceptible care transforms the abstract “Sport for Education” promise into a warm, supportive, and protected experience, which is the core psychological mechanism for building trust and loyalty. This validates the conclusions of Bhargava et al., demonstrating that a positive experience created by the organization directly translates into brand trust, a key mediating variable on the path to brand loyalty (18). Consequently, every aspect of event organization becomes a critical node in brand reputation management. Any organizational failure such as delayed medical response, course congestion, or unprofessional volunteer service would immediately erode the brand loyalty built through positive experiences. A well-structured organization with clear roles, responsibilities, and inter-departmental cooperation is the structural guarantee for the long-term success and trustworthiness of a university marathon brand.

### 5.3 Event culture: the ritualized practice of community cohesion

Once a trustworthy experiential platform is established, Event Culture (0.42) acts as a unique “converter,” translating the brand's abstract “Sport for Education” positioning into participants' value identification and collective sense of belonging through tangible symbols and rituals. The data show that participants place a very high value on material symbols that carry campus cultural elements, with “Event LOGO” (M = 4.21) and “Event medals, trophies” (M = 4.16) being particularly prominent. These are the tangible cultural symbols that every participant directly encounters and perceives, serving as emotional carriers for identity recognition and post-race self-validation.

Furthermore, the event culture elevates athletic competition into a “cultural ritual” that reinforces community identity, confirming the tenets of brand ontology theory. Students strongly identified with the spiritual values of the marathon in “developing adaptability for society” (M = 4.10), “courage to persist and challenge difficulties” (M = 4.05), and “enhancing personal self-confidence” (M = 4.02).Furthermore, this psychological transformation strengthens continuously alongside increased participation depth and cultural embeddedness. Such a characteristic is highly consistent with the immersive and ritualized attributes of the university marathon experience (38). This represents the micro-level process through which the “Sport for Education” brand positioning is realized. Through collective running, shared challenges, and mutual support, the abstract educational ideal is transformed into concrete, internalized character development and resilience building, uniting previously disparate individuals into a more emotionally cohesive community.

Notably, the data reveal a value discrepancy between different cultural symbols: the perceived value of the “Event medals” (M = 4.16) is significantly higher than that of “Event mascot, merchandise” (M = 3.70). This difference underscores the fundamental distinction between an earned identity and a purchased identity. The value of the medal is derived from the fact that it is an honor earned through sweat and perseverance a physical manifestation of a personal growth narrative that cannot be bought. It symbolizes the individual's successful passage through the collective ordeal of the race, thereby reinforcing their status and self-worth within the community. Merchandise, in contrast, is a passively acquired consumer symbol. In the university context, where personal growth and education are paramount, the value derived from hands-on practice and achievement is far more profound than that from commercial exchange. The university marathon functions not just as a cultural activity but as a modern rite of passage, complete with a preparatory phase (pre-race training), a liminal phase of trial and tribulation (the race itself), and a re-integration phase with a new status (a finisher, symbolized by the medal). It is this complete ritualistic process that powerfully translates the university's values perseverance, courage, and the indomitable sporting spirit into the internalized qualities of its participants.

### 5.4 Event services: the value co-creation pathway to brand loyalty

Although the path coefficient for Event Services (0.35) is the lowest of the four factors, this does not diminish its importance. On the contrary, it plays an indispensable role in the brand-building value chain, responsible for consolidating the positive experiences gained through activities, organization, and culture. In the experience economy, service is not a one-way provision from the organizer but an interactive process where the brand and participants co-create experience and generate value at every touchpoint (39).

The empirical data clearly point to the central role of digital touchpoints in this value co-creation process. “Online registration” received the highest score (M = 4.27) across all 25 items in the survey. This reveals a critical insight: for the “Z-generation” student audience, a seamless and efficient digital entry point is the starting line for their entire brand journey. Correspondingly high scores for “Event information delivery” (M = 3.99) and the “Sports app” (M = 3.89) construct an effective digital interaction matrix. These tools are more than just services; they are enabling platforms that allow participants to proactively access information, manage their race experience, and interact with the event and other participants, thereby actively co-creating the event's value.

Simultaneously, the tangible “hard services” during the race provide robust support. Participants' high approval of “On-course supplies”(4.01), “On-site services” (3.93), and “Medical and first aid” (3.87) reflects the university's genuine care for their well-being. These service touchpoints are the most heartfelt expressions of the “Sport for Education” philosophy, materializing abstract educational concern into perceptible safety and meticulous care, which are crucial for building deep brand loyalty.

It is noteworthy that “Official pacers” (3.60) received relatively lower attention. This is likely not an indicator of a service deficiency but rather a reflection that the core value proposition of a university marathon lies in broad participation, healthy experiences, and community interaction, rather than elite-level competition. This finding offers precise strategic guidance for resource allocation: organizers should prioritize investments in optimizing the universal digital experience and ensuring fundamental safety, rather than over-investing in high-level professional services needed by a minority of competitive runners. This once again reinforces the “Sport for Education” brand positioning, which is dedicated to the growth of all students, not just the athletic needs of a select few.

## 6 Conclusion and implications

### 6.1 Conclusion

Grounded in the core mission of “Sport for Education” in higher education, this study constructed and empirically validated a four-dimensional brand-building model comprising Event Activities, Event Organization, Event Culture, and Event Services. The study's primary theoretical contribution lies in using Structural Equation Modeling to reveal the distinct roles these elements play in brand formation and the intrinsic, hierarchical, and progressive logic that connects them. This has led to the proposition of a dynamic brand value generation path: one that begins with experience, is solidified by trust, elevated by identity, and culminates in co-creation.

Specifically, innovative and engaging Event Activities (0.55) serve as the primordial entry point for brand value, translating the abstract “educational” positioning into a tangible, embodied experience for participants, making it the absolute prerequisite for brand appeal. Building on this foundation, professional and reliable Event Organization (0.45) acts as the cornerstone of brand trust. Each safe and orderly execution of the event is a successful fulfillment of the brand promise, converting fleeting positive experiences into lasting brand trust and providing a structural guarantee for the brand's long-term development. Subsequently, a unique Event Culture (0.42) functions as a catalyst for brand value. Through shared symbols and collective rituals, it elevates the athletic activity into a cultural practice that reinforces the university community's identity, endowing the brand with deep emotional connections and enduring vitality. Finally, high-quality Event Services (0.35), present throughout the entire process, serve to consolidate brand reputation and loyalty. By facilitating positive interactions with participants at key touchpoints, these services transform participant satisfaction into brand loyalty, thus closing the loop of brand relationship building.

### 6.2 Practical implications

Based on these findings, the following strategic recommendations are offered to university administrators and event managers:

(1) From Single Event to Integrated Experience Matrix. Universities should re-envision the marathon not as a one-day race but as a semester- or even year-long comprehensive educational platform. By designing an activity matrix that integrates sensory, behavioral, emotional, and cognitive experiences—seamlessly embedding knowledge sharing and skill development into the event's full lifecycle—the abstract positioning of “Sport for Education” can be transformed into tangible, multidimensional value for participants.
(2) From Backstage Support to Frontstage Communication. Organizational management should not be treated as passive, behind-the-scenes support. Instead, it should be proactively and transparently communicated as proof of the brand's professionalism. Transforming intangible organizational competence into a perceptible sense of professionalism, safety, and care is fundamental to building brand trust.
(3) From Static Symbols to Dynamic Rituals. The focus of brand building should shift from the static display of symbols to the dynamic creation of atmosphere. Emphasis should be placed on celebrating personal achievement (the story behind the medal) rather than on commercialization (merchandise). By designing collective, ritualistic moments, the event can be elevated into a cultural practice that powerfully reinforces the university community's identity.
(4) From Service Provision to Value Co-Creation. Given the high satisfaction with digital touchpoints, administrators should prioritize investment in developing more interactive digital tools. These tools can empower participants to take an active role in shaping the event experience, transforming satisfied participants into loyal brand advocates and active brand promoters.

## 7 Limitations and future research directions

While this study provides a robust empirical foundation for the brand building of university marathons, several limitations warrant acknowledgment. First, the data in this study were collected exclusively from a single university. Although the selected case is highly representative, the generalizability of the research findings to higher education institutions of different types and geographic regions remains to be rigorously tested. Accordingly, future research should expand the sample scope and conduct cross-institutional comparative studies to strengthen the external validity of the conclusions.

Second, future research may adopt a longitudinal design to track the trajectories of changes in participants' brand perception, brand identification, and brand loyalty following repeated engagement with the event. In addition, more diverse research perspectives can be incorporated. Specifically, subsequent studies can explore the brand risk management and green sustainable development pathways of university marathons, as well as their tangible impacts on long-term brand performance indicators including alumni giving and enrollment attractiveness. This line of inquiry will ultimately facilitate the development of a more comprehensive, multi-dimensional theoretical framework for campus sporting event branding.

## Data Availability

The original contributions presented in the study are included in the article/Supplementary Material, further inquiries can be directed to the corresponding author.
